# Geographical Variations in the Environmental Determinants of Physical Inactivity among U.S. Adults

**DOI:** 10.3390/ijerph14111326

**Published:** 2017-10-31

**Authors:** Ruopeng An, Xinye Li, Ning Jiang

**Affiliations:** 1 Department of Kinesiology and Community Health, University of Illinois at Urbana-Champaign, Champaign, IL 61820, USA; ran5@illinois.edu; 2 Bloomberg School of Public Health, Johns Hopkins University, Baltimore, MD 21205, USA; xli175@jhmi.edu; 3 School of Economics and Resource Management, Beijing Normal University, Beijing 100875, China

**Keywords:** physical inactivity, environmental quality, geographical variation, geographically weighted regression

## Abstract

Physical inactivity is a major modifiable risk factor for morbidity, disability and premature mortality worldwide. This study assessed the geographical variations in the impact of environmental quality on physical inactivity among U.S. adults. Data on county-level prevalence of leisure-time physical inactivity came from the Behavioral Risk Factor Surveillance System. County environment was measured by the Environmental Quality Index (EQI), a comprehensive index of environmental conditions that affect human health. The overall EQI consists of five subdomains—air, water, land, social, and built environment. Geographically weighted regressions (GWRs) were performed to estimate and map county-specific impact of overall EQI and its five subdomains on physical inactivity prevalence. The prevalence of leisure-time physical inactivity among U.S. counties was 25% in 2005. On average, one standard deviation decrease in the overall EQI was associated with an increase in county-level prevalence of leisure-time physical inactivity by nearly 1%. However, substantial geographical variations in the estimated environmental determinants of physical inactivity were present. The estimated changes of county-level prevalence of leisure-time physical inactivity resulted from one standard deviation decrease of the overall EQI ranged from an increase of over 3% to a decrease of nearly 2% across U.S. counties. Analogous, the estimated changes of county-level prevalence of leisure-time physical inactivity resulted from one standard deviation decrease of the EQI air, water, land, social, and built environment subdomains ranged from an increase of 2.6%, 1.5%, 2.9%, 3.3%, and 1.7% to a decrease of 2.9%, 1.4%, 2.4%, 2.4%, and 0.8% across U.S. counties, respectively. Given the substantial heterogeneities in the environmental determinants of physical inactivity, locally customized physical activity interventions are warranted to address the most concerning area-specific environmental issue.

## 1. Introduction

Physical inactivity is a major modifiable risk factor for morbidity, disability and premature mortality worldwide [1]. Physical activity promotion serves as a key public health strategy [2]. However, four in five American adults fail to meet guidelines-recommended physical activity levels [3]. While traditional perspective mainly blamed individuals themselves for their sedentary lifestyle, increasing attention has been shifted to the environmental determinants of health behavior—how political, economic, ecological, and social contexts in which people are born, live, work and age impact their physical activity [4]? For instance, availability and proximity of parks, bike lanes and recreational facilities has been consistently linked to increased physical activity and active commuting [5]; whereas neighborhood crime rate is inversely associated with outdoor activities and sports among local residents [6].

One major challenge in understanding the complex relationship between environment and physical activity is that both factors vary substantially across the U.S. While spatial variations in the prevalence of physical inactivity have been consistently documented [7], little is known regarding the potential spatial heterogeneities of the environmental determinants of physical inactivity—whether and to what extent spatial variations in physical inactivity are associated with spatial variations in environmental attributes. Examining geographical variations in the relationship between environment and physical activity could help customize and prioritize population-level policy interventions and improve their effectiveness in reducing sedentary behavior and promoting a healthier lifestyle. For example, if it is found that high crime rate, air pollution, and lack of parks and bike lanes are the respective key driver of physical inactivity in three different geographical areas, instead of launching a universal intervention across all three locations, customized interventions of law enforcement, emission regulation, and infrastructure enhancement should be carefully evaluated and implemented in each area.

Conventional models are mostly global, which assume and estimate a single effect that remains constant across geographical locations. In this study, we assessed the geographical variations in the environmental determinants of physical inactivity among U.S. adults using geographically weighted regression (GWR). GWR relaxes the assumption of statistical independence between observations and spatial stationarity, and can provide local estimate specific to each geographical area of interest [8]. The unique features of GWR make it well suited to examine the differential environmental impacts on physical activity across the nation.

## 2. Methods

### 2.1. Data

Data on county-level prevalence of leisure-time physical inactivity came from the Centers for Disease Control and Prevention (CDC)’s County Data Indicators (CDIs) [9]. The CDIs are estimated based on the annual survey data collected from the Behavioral Risk Factor Surveillance System (BRFSS). BRFSS is the nation’s primary system of health-related repeated cross-sectional telephone surveys that collect state data about U.S. residents regarding their health-related risk behaviors, chronic conditions, and use of preventive services. BRFSS questionnaires, sampling design and survey datasets can be found elsewhere [10]. Since 2001, the question regarding leisure-time physical inactivity in BRFSS is, “During the past month, other than your regular job, did you participate in any physical activities or exercises such as running, calisthenics, golf, gardening, or walking for exercise?”. Leisure-time physical inactivity was identified from answers of “no” to this question.

Environmental quality is the characteristics and properties of the environment that impact human beings and other organisms [11]. Developed by the Environmental Protection Agency (EPA), Environmental Quality Index (EQI) is a comprehensive measure of county-level environmental conditions that affect human health [11,12]. Five subdomains contribute to environmental quality: air, water, land, built, and social environments. The air subdomain is constructed based on two data sources that document monitoring, emissions, and modeled estimates of criteria and hazardous air pollutants. The water subdomain is constructed based on nine data sources that document drinking water quality, public water supply, draught, and pollutants in rainfall and recreational water. The land subdomain is constructed based on 12 data sources that document agriculture, industrial facilities, geology and mining, and land cover. The built subdomain is constructed based on four data sources that document traffic and transit, pedestrian safety, access to physical activity, food environment, and school environment. The social subdomain is constructed based on three data sources that document county-level sociodemographics (e.g., percent of non-English speakers, and median household income), mean number of violent crimes per capita, and housing conditions (e.g., percent of renter occupied, percent of vacant units, and median number of rooms per house). Each domain forms its own separate index, and the overall EQI is the sum of scores from all five subdomains. Both the overall EQI and its five subdomains are standardized to have a mean of zero and a standard deviation of one. Therefore, a positive EQI denotes a higher-than-average environmental quality, and a negative EQI denotes a lower-than-average environmental quality. No specific cutoffs for the standardized EQI scores are provided by the EPA. In this study, we adopted a continuous measure of EQI scores, and no specific cutoffs were used. Detailed information regarding the EQI construction and relevant data sources can be found elsewhere [11,12].

The EQI measures were constructed based on the data sources collected during 2000–2005. The EPA is currently working to update the EQI based on more recent data but the new indices have yet been available [11]. Therefore, this study used the 2005 data for physical inactivity prevalence and all other county-level characteristics.

The following county-level characteristics that were likely to correlate with residents’ physical activity level were adjusted in regression analyses. Annual average daily maximum temperature (°F), daily sunlight (KJ/m^2^) and daily precipitation (mm) came from the CDC WONDER systems [13]. Population density (1000 persons per square mile), percentage of racial/ethnic minorities, percentage of high school and lower education, and percentage of households below the federal poverty level came from the U.S. Census Bureau [14]. Data on annual unemployment rate came from the U.S. Bureau of Labor Statistics [15].

### 2.2. Statistical Analysis

We performed GWR to examine the geographical variations in the environmental determinants of physical inactivity in the contiguous U.S. Conventional regressions such as the Ordinary Least Squares (OLS) are a global model, which assumes that study subjects residing in different neighborhoods are independent of each other [16]. However, spatial features and their associated data values are often clustered together in space (i.e., positive spatial autocorrelation) or dispersed (i.e., negative spatial autocorrelation), which violates the assumption of statistical independence in the OLS. Moreover, OLS estimates a single set of associations between the dependent variable and the independent variables, which implies spatial stationarity of the relationship. However, the characteristics of a particular area may impact the direction and magnitude of the relationship, which can deviate from the global estimate. GWR relaxes the assumptions of statistical independence and spatial stationarity and produces a range of area-specific coefficients. GWR instantaneously performs many regressions so that there is one regression per spatial data point (e.g., county). Observations closer to a particular data point will have a higher weight than those farther away. These distinct features of GWR makes it particularly suitable to assess the spatial variations in the relationship between local environment and outcomes of interest.

We followed the three steps below in statistical analysis. First, we conducted two OLS regressions. The dependent variable in both models was county-level percentage of leisure-time physical inactivity. The key independent variable in the first OLS regression was the overall EPI, which was standardized to have a mean of zero and standard deviation of one (i.e., EPI z-score). The key independent variables in the second OLS regression were the five standardized EPI subdomains—air, water, land, built, and social environment. Both OLS regressions controlled for county-level characteristics including daytime temperature, sunlight, precipitation, population density, percentage of racial/ethnic minorities, percentage of high school and lower education, percentage of households below the federal poverty level, and unemployment rate. Second, we calculated the Moran’s I of the residuals estimated from the OLS regressions across U.S. counties. As a measure of spatial autocorrelation, a large and statistically significant Moran’s I would suggest the presence of substantial geographical variations in physical inactivity rates that were not explained by the OLS regressions, which justified the use of GWR. Third, we performed two GWRs, with the overall EPI and the five EPI subdomains as their respective key independent variable(s). Bothe GWRs controlled for county-level characteristics. We then calculated the Moran’s I of the residuals estimated from the GWRs across U.S. counties. If the GWRs could better model geographical variations of physical inactivity rates than the OLS regressions, the Moran’s I of the residuals estimated from the GWRs should be substantially reduced in comparison to that from the OLS regressions. We also compared the R-squared between the GWRs and the OLS regressions. If the GWRs fitted the data substantially better than the OLS regressions did, the R-squared from the GWRs should be noticeably larger than that from the OLS regressions.

OLS regressions were performed using Stata 14.2 SE version (StataCorp, College Station, TX, USA). GWRs were performed using GWR4.09. U.S. county maps were constructed using ArcGIS10.5 (ESRI, Redlands, CA, USA).

### 2.3. Ethical Approval

This study used publicly available county-level aggregate data and involved no human subjects. Therefore, the study was exempted from human subject review by the corresponding author’s university institutional review board.

## 3. Results

Table 1 summarizes county-level characteristics. The prevalence of leisure-time physical inactivity among U.S. counties averaged 25% in 2005. Annual average daily maximum temperature was 65.3 °F, average daily sunlight 16.3 KJ/m^2^, and average daily precipitation 2.6 mm. County-average population density was 250 persons per square mile. Racial/ethnic minorities accounted for 13.1% of county population, education of high school and lower 17.3%, and household below poverty line 15.3%. Annual unemployment rates averaged 5.4% across U.S. counties in 2005.

Table 2 and Table 3 report the estimated impacts of environmental quality on physical activity based on the OLS regressions. One standard deviation decrease in the overall EQI was found to be associated with an increase in county-level prevalence of leisure-time physical inactivity by nearly 1% (95% confidence interval (CI) = 0.81%, 1.15%) (Table 2). One standard deviation decrease in the EQI water, social, and built environment subdomains were associated with an increase in county-level prevalence of leisure-time physical inactivity by 0.41% (95% CI = 0.28%, 0.54%), 0.37% (95% CI = 0.03%, 0.70%), and 0.65% (95% CI = 0.51%, 0.79%), respectively (Table 3). EQI air and land subdomains were not found to be associated with county-level physical inactivity (*p*-values ≥ 0.05). Annual average daily maximum temperature and precipitation, percent of racial/ethnic minorities, education of high school and lower, and households below the federal poverty level, and annual unemployment rate were positively associated with county-level physical inactivity; whereas annual average daily sunlight was negatively associated with physical inactivity.

Table 4 and Table 5 report the estimated impacts of environmental quality on physical activity based on the GWRs. Substantial geographical variations in the estimated environmental determinants of physical inactivity were present. The estimated changes of county-level prevalence of leisure-time physical inactivity resulted from one standard deviation decrease of the overall EQI ranged from an increase of over 3% to a decrease of nearly 2% across U.S. counties (Table 4). Statistically significant inverse associations between the overall EQI and physical inactivity rate occupied over 20% of all county-specific coefficient estimates, whereas statistically significant positive associations accounted for merely 1%. The estimated changes of county-level prevalence of leisure-time physical inactivity resulted from one standard deviation decrease of the EQI air, water, land, social, and built environment subdomains ranged from an increase of 2.6%, 1.5%, 2.9%, 3.3%, and 1.7% to a decrease of 2.9%, 1.4%, 2.4%, 2.4%, and 0.8% across U.S. counties, respectively (Table 5). Statistically significant inverse associations between the EQI air, water, land, social, and built environment subdomains and physical inactivity rate occupied 6%, 20%, 12%, 29%, and 21% of all county-specific coefficient estimates, whereas statistically significant positive associations accounted for 16%, 14%, 5%, 1%, and 1%, respectively.

**Figure 1:**
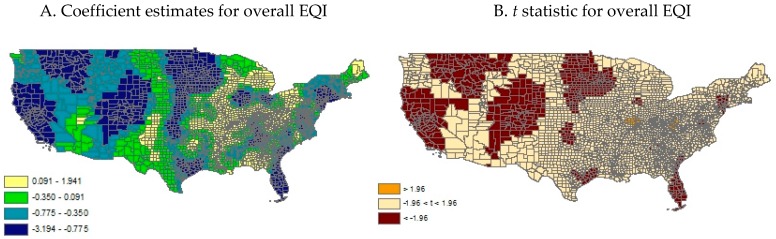
Estimated county-specific impacts of standardized overall EQI on physical inactivity prevalence using GWR.

**Figure 2:**
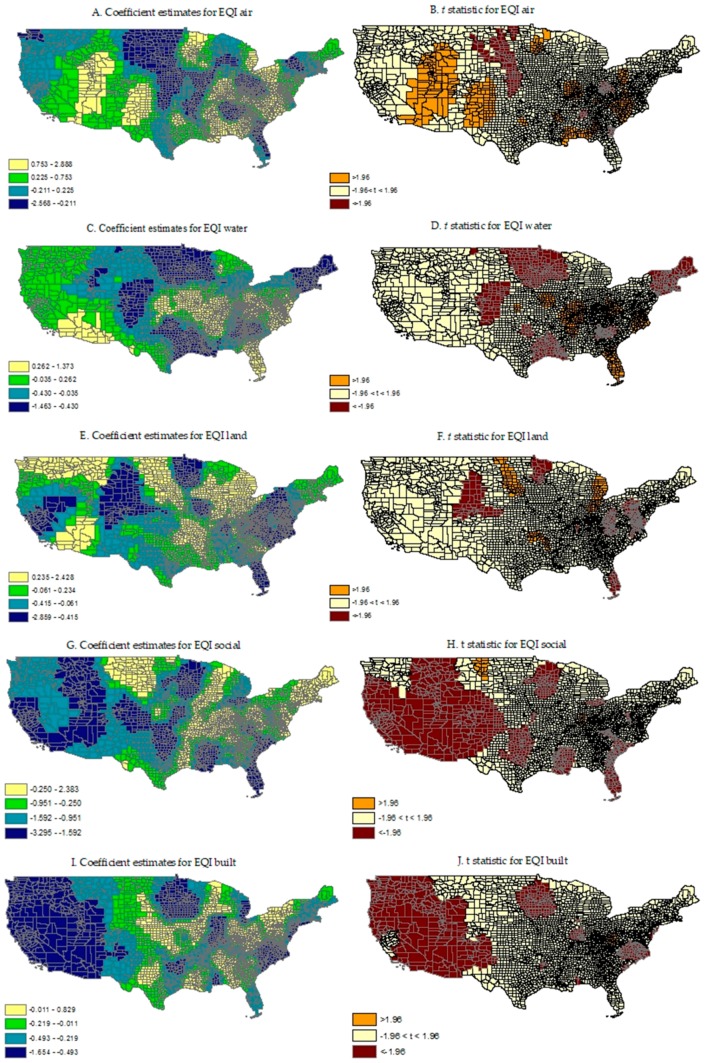
Estimated county-specific impacts of standardized EQI subdomains on physical inactivity prevalence using GWR.

Figure 1 and Figure 2 map the estimated county-specific impact of the overall EQI and its subdomains on the prevalence of leisure-time physical inactivity using GWRs.

Moran’s I of the residuals estimated from the OLS regressions were 0.092 (95% CI = 0.090, 0.093) for the overall EQI and 0.089 (95% CI = 0.088, 0.090) for the five EQI subdomains, denoting substantial geographical variations across U.S. counties that were not explained by these global models. In comparison, Moran’s I of the residuals estimated from the GWRs were reduced to −0.0003 (95% CI = −0.001, 0.001) for the overall EQI and 0.0002 (95% CI = −0.001, 0.001) for the five EQI subdomains. The *R*-squared increased from 0.58 based on the OLS regression to 0.87 based on the GWR with the overall EQI as the key independent variable. Analogously, the *R*-squared increased from 0.58 based on the OLS regression to 0.86 based on the GWR with the five EQI subdomains as the key independent variables.

## 4. Discussion

This study assessed the geographical variations in the environmental determinants of physical inactivity among U.S. adults. Prevalence of leisure-time physical inactivity was matched to the overall EQI and its five subdomains by residential county. GWRs were performed to estimate county-specific associations between environmental quality and physical inactivity rate, adjusting for various county-level characteristics. Substantial geographical variations in the estimated environmental determinants of physical inactivity were revealed.

In general, this study confirmed an inverse relationship between environmental quality and physical inactivity documented in the previous literature [17,18,19]. However, the effect magnitude is quite moderate—one standard deviation decrease in the overall EQI on average led to an increase in county-level prevalence of leisure-time physical inactivity by approximately 1%. However, the negative impact of compromised environmental quality on physical activity was not uniformly distributed across geographical areas but concentrated in about 20% of U.S. counties. Those counties tended to cluster within a few states such as California, Florida, Montana, North Dakota and South Dakota. Among the five environmental subdomains, built and social environment tended to exert the largest impacts. This coincides with the body of evidence that predominantly concentrates upon the influence of built environment and neighborhood social environment on physical activity engagement [18,20]. Moreover, these two EQI subdomains shared fairly similar geographical variations in their estimated impact—the counties most influenced by built and social environment tended to reside in a few states such as Minnesota, Montana, Utah, Colorado, and California. Accessibility and quality of drinking and recreational water, as measured by the EQI water subdomain, and to a lesser extent, air and land conditions, were also found to link to physical inactivity. However, the geographical distributions of their respective impact differed substantially from each other and also noticeably diverged from their counterparts of built and social environment.

Given the substantial heterogeneities in the estimated environmental determinants of physical inactivity, customized policy interventions that address specific and most concerning environmental issue in a local area could be more effective (and cost-effective) than a nationwide universal intervention. While evidence-based interventions addressing various environmental attributes have provided a rich set of policy options, local government and stakeholders should carefully assess their specific situation and choose the option that best meet their individual needs [21]. For instance, in an area where air pollution is the major deterrent to physical activity, pollution control policies should be prioritized over park and bike lane construction in order to promote a more active lifestyle. Moreover, given limited resources, local government should prioritize their policy options based on a cost-benefit calculation. While some of the environmental determinants are more difficult and/or expensive to change (e.g., poverty, landscaping), others are relatively less costly and/or resource consuming (e.g., air and water quality monitoring). Government may invest more on environmental interventions that provide a larger marginal return per dollar spent.

GWR has been increasingly widely used to examine spatially varying patterns and relationships in both chronic conditions (e.g., diabetes) and communicable diseases (e.g., malaria) [16,22]. The major advantage of GWR over a global model such as OLS is that it explicitly models spatial autocorrelation and produces location-specific estimates (as well as their respective uncertainty measures, e.g., standard error, CI, and *p*-value). Spatial dependency is a testable hypothesis (e.g., using Moran’s I), and in absence of it, GWR is reduced to an OLS regression. In this study, the Moran’s I of the residentials were substantially reduced and became statistically non-significant when replacing OLS with GWR, suggesting that most, if not all, the spatial heterogeneity in the OLS-estimated environmental determinants of physical inactivity could be accounted for by GWR. In addition, the substantial increase in the R-squared also reflects the improved goodness of fit of GWR relative to OLS.

This study is the first that examined geographical variations in the environmental determinants of physical inactivity among U.S. adults. Measures on environmental quality were comprehensive and constructed based on a large pool of authoritative data sources. GWR illustrated spatially varying relationship between environmental attributes and physical inactivity that could not be revealed using conventional regression approaches. Nevertheless, a few limitations of the study warrant caution. Leisure-time physical activity in the BRFSS was based on self-report and prone to recall problem and social desirability bias [23]. The sampling design of BRFSS enables state-representative health indicator estimates, but the representativeness in general does not extend to local estimates below the state level (e.g., county or city). More recent EQI and its subdomains based on 2010 Census data are under construction but have yet been available, which prevented us from using more recent BRFSS surveys. Arguably, 12 years have passed since the EQI and physical inactivity data were collected, and the estimated county-specific relationships between EQI and physical inactivity might have changed over time. However, in the absence of up-to-date data, no reliable projections could be made regarding the long-term trajectory for the local variations of physical inactivity in relation to environmental quality. The EQI measures are comprehensive but abstract, so that we are unable to differentiate the influence of each specific contributing factor (e.g., park versus bike lane, crime versus housing). This limits our ability to provide more specific county-level policy recommendations. Upon the availability of more recent physical inactivity and EQI data, future studies may adopt a longitudinal study design and examine the change of physical inactivity patterns in response to the change of environmental quality across U.S. counties.

## 5. Conclusions

This study assessed the environmental determinants of physical inactivity among U.S. adults. In general, an inverse relationship was identified between environmental quality and physical inactivity. Substantial geographical variations in the estimated relationship were present. The estimated changes of county-level prevalence of leisure-time physical inactivity resulted from one standard deviation decrease of the overall EQI ranged from an increase of over 3% to a decrease of nearly 2% across U.S. counties. Given the substantial heterogeneities in the estimated environmental determinants of physical inactivity, locally customized physical activity interventions are warranted to address the most concerning area-specific environmental issue.

## Figures and Tables

**Table 1:** Summary statistics of U.S. counties.

Variable	Mean	SD	Median
Physical inactivity (%)	25.25	5.11	24.90
Standardized EQI	0	1	0.07
Standardized EQI air	0	1	0.01
Standardized EQI water	0	1	0.23
Standardized EQI land	0	1	0.19
Standardized EQI social	0	1	0.01
Standardized EQI built	0	1	0.16
Average daily maximum temperature (°F)	65.31	8.65	64.94
Average daily sunlight (KJ/m^2^)	16.25	1.49	16.05
Annual precipitation (mm)	2.57	0.94	2.58
Population density (1000 persons/mi^2^)	0.25	1.70	0.05
Racial/ethnic minorities (%)	13.08	15.68	6.21
Education less than high school (%)	17.33	7.86	15.61
Poverty (%)	15.34	6.52	14.20
Unemployment rate (%)	5.41	1.78	5.10

**Table 2:** Estimated effect of overall environmental quality index (EQI) on county-level prevalence of physical inactivity using OLS.

Variable	Coefficient	SE	*t*-Statistic	*p*-Value	VIF
Intercept	24.118	0.816	29.548	0.000	
Standardized EQI	−0.979	0.084	−11.590	0.000	1.83
Average daily maximum temperature (°F)	0.338	0.013	26.062	0.000	3.51
Average daily sunlight (KJ/m^2^)	−1.717	0.068	−25.122	0.000	2.89
Annual precipitation (mm)	0.686	0.070	9.824	0.000	1.20
Population density (1000 persons/mi^2^)	−0.034	0.036	−0.930	0.352	1.07
Racial/ethnic minorities (%)	0.0379	0.005	7.503	0.000	1.75
Education less than high school (%)	0.195	0.013	15.039	0.000	2.90
Poverty (%)	0.047	0.017	2.799	0.005	3.39
Unemployment rate (%)	0.117	0.042	2.763	0.006	1.59

**Table 3:** Estimated effects of environmental quality index (EQI) subscales (i.e., air, water, land, built, and social environment) on county-level prevalence of physical inactivity using OLS.

Variable	Coefficient	SE	*t*-Statistic	*p*-Value	VIF
Intercept	23.625	0.938	25.187	0.000	
Standardized EQI air	0.093	0.095	0.974	0.330	2.60
Standardized EQI water	−0.408	0.065	−6.324	0.000	1.18
Standardized EQI land	−0.122	0.076	−1.608	0.107	1.62
Standardized EQI social	−0.365	0.171	−2.133	0.033	8.27
Standardized EQI built	−0.652	0.072	−9.018	0.000	1.48
Average daily maximum temperature (°F)	0.310	0.014	21.489	0.000	4.40
Average daily sunlight (KJ/m^2^)	−1.558	0.072	21.513	0.000	3.28
Annual precipitation (mm)	0.559	0.078	7.161	0.000	1.51
Population density (1000 persons/mi^2^)	−0.068	0.037	−1.848	0.064	1.11
Racial/ethnic minorities (%)	0.032	0.005	6.198	0.000	1.86
Education less than high school (%)	0.188	0.014	13.114	0.000	3.59
Poverty (%)	0.070	0.023	3.032	0.002	6.41
Unemployment rate (%)	0.094	0.044	2.163	0.031	1.69

**Table 4:** Estimated effect of overall environmental quality index (EQI) on county-level prevalence of physical inactivity using geographically weighted regression (GWR).

Variable	Coefficient		Proportion of Counties by *t*-Statistic		
	Min	Max	*t* ≤ −1.96	−1.96 < *t* < 1.96	*t* ≥ 1.96
Intercept	−151.200	106.800	0.088	0.507	0.405
Standardized EQI	−3.194	1.941	0.203	0.784	0.013
Average daily maximum temperature (°F)	−1.070	3.885	0.067	0.676	0.257
Average daily sunlight (KJ/m^2^)	−10.740	13.790	0.250	0.687	0.063
Annual precipitation (mm)	−5.634	8.604	0.138	0.719	0.143
Population density (1000 persons/mi^2^)	176.800	2.883	0.379	0.621	0.0003
Racial/ethnic minorities (%)	−0.298	0.266	0.022	0.627	0.351
Education less than high school (%)	−0.354	0.659	0.020	0.420	0.560
Poverty (%)	−0.359	0.416	0.056	0.868	0.076
Unemployment rate (%)	−1.156	1.752	0.096	0.696	0.208

**Table 5:** Estimated effect of overall environmental quality index (EQI) on county-level prevalence of physical inactivity using geographically weighted regression (GWR).

Variable	Coefficient		Proportion of Counties by *t*-Statistic		
	Min	Max	*t* ≤ −1.96	−1.96 < *t* < 1.96	*t* ≥ 1.96
Intercept	−83.020	99.490	0.08	0.379	0.541
Standardized EQI air	−2.568	2.888	0.062	0.774	0.164
Standardized EQI water	−1.464	1.372	0.201	0.661	0.138
Standardized EQI land	−2.859	2.428	0.117	0.830	0.053
Standardized EQI social	−3.295	2.383	0.288	0.700	0.012
Standardized EQI built	−1.654	0.828	0.205	0.785	0.010
Average daily maximum temperature (°F)	−0.769	3.071	0.067	0.612	0.321
Average daily sunlight (KJ/m^2^)	−9.869	9.268	0.324	0.614	0.062
Annual precipitation (mm)	−4.077	5.126	0.202	0.665	0.133
Population density (1000 persons/mi^2^)	−112.000	1.236	0.524	0.476	0.000
Racial/ethnic minorities (%)	−0.210	0.236	0.022	0.572	0.407
Education less than high school (%)	−0.249	0.611	0.023	0.500	0.477
Poverty (%)	−0.278	0.361	0.082	0.906	0.011
Unemployment rate (%)	−0.885	1.227	0.141	0.594	0.265
